# Treatment decision making in psychiatry: Formulating patients’ perspectives in outpatient psychiatric consultations

**DOI:** 10.3389/fpsyg.2023.1144500

**Published:** 2023-03-27

**Authors:** Xueli Yao, Xiaoning Zhang

**Affiliations:** ^1^School of Foreign Languages, Qingdao Agricultural University, Qingdao, China; ^2^Shandong Provincial Medical Association, Jinan, China

**Keywords:** treatment decision making, psychiatric outpatient consultation, formulation, conversation analysis, talk-in-interaction

## Abstract

Seeking and understanding patients’ values and preferences is one of the essential elements in shared decision making, which is associated with treatment adherence in psychiatry. However, negotiating treatment in psychiatric contexts can be challenging with patients whose ability to evaluate treatment recommendations rationally may be impaired. This article attempts to examine a conversational practice that psychiatrists use to deal with patients’ views and perspectives by formulating what the patients have said related to treatment. Taking the naturally occurring, face-to-face outpatient psychiatric consultations as the data, the present study uses conversation analysis (CA) as a method to demonstrate in a fine-grained detail what functions formulations of patients’ perspectives serve in psychiatric contexts. We found that by eliciting patients’ views and perspectives toward treatment, this type of formulation is not only used to achieve mutual understanding and establish the grounds for treatment decisions, but may also be used to challenge the legitimacy of patients’ position, steering treatment decision to the direction preferred by the psychiatrists. We argue that in the process of treatment decision making, psychiatrists do not simply impose their perspectives upon the patients, instead, they attempt to achieve consensus with patients by balancing their institutional authority and orientation to the patients’ perspectives. Data are in Chinese with English translation.

## 1. Introduction

In the field of psychiatry, as in many areas of medicine, there has been a trend toward patient-centered care that emphasizes shared decision making with patients in planning and enacting treatment regimens (Quirk et al., 2012; Angell and Bolden, 2015; Bolden and Angell, 2017; Thompson and McCabe, 2018). It highlights the importance of understanding and seeking patients’ values, needs and treatment preferences, which is associated with treatment adherence and regarded as a cornerstone of successful treatment in mental health care (Thompson and McCabe, 2012; McCabe et al., 2013). Despite the significance of patient-centered practice, recent research suggests that the shared decision-making model is only partially actualized in medical encounters in general and in psychiatry in particular (Seale et al., 2006; Adams et al., 2007; Woltmann and Whitley, 2010).

It seems that two issues are relevant in the negotiation of treatment decision making: first, who should make the decision, and second, what knowledge should be taken as the basis for the decision. The first issue falls within the domain of deontics and the second epistemics (Landmark et al., 2015). Epistemic domain relates to a person’s knowledge and personal experiences (Heritage, 2012, 2013). In the area of medicine, patients have primary rights to knowledge about their experiences of illness and preferences while doctors have professional authority (Landmark et al., 2015). Deontic domain on the other hand is concerned with participants’ rights to determine future actions (Lindström and Weatherall, 2015). There is a struggle between doctors’ epistemics of expertise and patient’s epistemics of subjective experience: if the claim is grounded in doctors’ expertise domain, the doctors would assert their deontic authority; while if the claim belongs to patient’s epistemic domain, the patients’ deontic rights would be invoked (Ekberg and LeCouteur, 2015).

It has been pointed out that the implementation of shared decision making in mental health treatment is particularly delicate and challenging (Bolden and Angell, 2017). On the one hand, although exercise of authority does not necessarily connote a paternalistic model of treatment decision (Angell and Bolden, 2015), the asymmetry in medical encounters does have some constraint on the doctor-patient collaboration in decision making (Pilnick and Dingwall, 2011). In medical encounters, it is taken for granted that patients seek medical expertise to deal with their problems and doctors are socialized to exercise authority. In this sense, patient-centered care may be considered undermining this taken-for-granted social order. On the other hand, the situation is complicated in psychiatric contexts by the fact that some patients with severe mental disorders, such as schizophrenia, may have poor sense of reality or poor awareness of their illness leading to resistance to treatment (Thompson and McCabe, 2018). With the patients who lack ability to evaluate treatment options rationally, psychiatrists may feel obliged to adopt a paternalistic approach, persuading or even coercing them to accept the treatment proposals (Brodwin, 2013).

The discrepancy between the prominence of shared decision making and the practice of treatment management in reality arouses the attention from scholars and researchers alike. The vast health communication studies on shared decision making have focused on the development of conceptual models and guidelines for conducting communication associated with shared decision making and patient involvement (Elwyn et al., 2000; Krupat et al., 2006; Makoul and Clayman, 2006; Clayman et al., 2012); however, they are less helpful in saying how it is to be done *in situ* in that the interactional realization of such a model is a far more complex issue. Therefore, it is of great importance to carry out fine-grained empirical analyses of talk-in-interaction in the course of authentic encounters.

Recently, as compensation for this deficiency, there is a small but growing amount of literature concerning conversation analytic studies on medical decision making. Through fine-grained observational analyses of naturally occurring, face-to-face talk-in-interaction in psychiatry, these studies show that psychiatrists may attempt to take into consideration patients’ needs and involvement of patients in treatment decision making in mental health settings. For instance, in their work on therapeutic group care for adults with mental disorders, Mortari and Pino (2014) demonstrate that mental health professionals may recur to some conversational practices to mobilize clients’ cooperation and promote their medication compliance. Kushida and Yamakawa (2015) point out that psychiatrists design their treatment recommendations so as to fit the patient’s perspectives and elicit an acceptance of the treatment proposal. Psychiatrists may justify their medication decisions by drawing on their professional expertise on the one hand and the patients’ concerns and needs on the other (Angell and Bolden, 2015). The present article contributes to this small but growing body of literature on treatment decision making in psychiatry by examining one conversational practice psychiatrists recurrently use to deal with patients’ perspectives in authentic psychiatrist-patient encounters, namely, formulation. By focusing on psychiatrists’ formulation of patients’ views and preferences toward treatment, we explore how the formulation is utilized to achieve some of the interactional work involved in treatment decision making in psychiatry.

In what follows, we begin by briefly introducing the term *formulation* and the previous research on how this sort of conversational practice is related to shared decision making in medical settings. This will form the basis for our analysis of the present study, presented in the section “Results.”

## 2. The formulation in previous research

Formulation, described as summaries or paraphrases of others’ talk (Heritage and Watson, 1979; Heritage, 1985), is a common conversational practice in institutional settings. It involves an assertion of some understanding of the previous speaker’s talk (Heritage and Watson, 1979) and works to solicit confirmation from the speaker. Formulation opens a sequential slot in which the participant may accept/confirm or reject/disconfirm the formulation in the next turn (Heritage, 1985; Drew, 2003).

It is further pointed out that although formulation is about summarizing, paraphrasing, or giving the gist or upshot of what has been said in the previous turns of talk, it is in fact not entirely neutral and is rarely undertaken for its own sake; rather, it is selective and tendentious in nature (Hutchby, 2005). It allows the current speaker to either select, delete, transform, explain or extend the previous speaker’s words and at the same time preserves the relevant features of the previous utterance (Antaki, 2008), which may make it serve specific functions in a variety of institutional settings (Weiste, 2016).

Conversation analytic study of formulation dates from Davis’ (1986) exploration of it in psychotherapy. She displayed that formulation may be deployed by the therapist to transform the clients’ initial version of their troubles into a psychological problem. Ever since Davis’ work, formulation has become a research topic that remains in the CA researchers’ agenda (e. g. Hak and de Boer, 1996; Gafaranga and Britten, 2004; Barnes, 2007; Antaki, 2008; Landmark et al., 2016). For instance, Antaki et al. (2005) and Antaki (2008) further find out that in psychotherapy, formulation of client’s talk may serve the therapist interest in a variety of ways, such as getting clients’ history, shaping symptoms and closing down troubles.

Drew (2003) has pointed out that formulations are associated with core tasks of different institutional settings and serve different functions depending on the types of the setting. In psychiatric care, seeking and discussing patients’ preferences and values seems to be one of these core tasks. As formulation is about summarizing, paraphrasing, or giving the gist or upshot of what has been said in the previous turns of talk, it may serve the function of fostering mutuality in medical settings (Gafaranga and Britten, 2004). In addition, by inviting confirmation from the patients, the formulation does more than seeking and checking shared understanding of patients’ perspectives, but also allows patients an opportunity to elaborate, repair or even deny the formulation, thus making the patients exert more agency in the process of decision making.

Thus, in the present study, we focus on formulations of patients’ treatment perspectives in a type of psychiatric care, namely, routine outpatient psychiatric consultations. Specifically, we show how and in what interactional environments this type of formulation is used in the process of treatment decision making.

In China, psychiatric outpatient clinics are part of psychiatric services where a broad range of mental disorders, ranging from chronic to acute ones, are diagnosed, consulted and treated. In routine psychiatric outpatient consultations, patients, usually those with severe and prolonged mental disorders, meet their psychiatrists at regular intervals (generally once a month) to have their stability monitored, medication checked and mental health condition evaluated. According to the evaluation, treatment recommendations are presented and discussed, mainly focusing on medication management.

## 3. Data and methods

The data for the present study consist of 35 audio-recorded routine consultations in a psychiatric outpatient clinic in China, collected by the first author of the present article from 2017 to 2019 with informed consents from all the participants: five psychiatrists and 35 patients. The psychiatrists, one female and four males, are expert psychiatrists aging from 45 to 76, whose expertise is in the diagnosis and treatment of serious mental illness, such as schizophrenia, bipolar disorder and neurosis. It is stipulated in this clinic that only expert psychiatrists be qualified to provide outpatient services and it explains why only expert psychiatrists are included in our data.

All the 35 patients, 14 females and 21males, aging from 21 to 68, meet CCMD-3 (a Chinese version of ICD-10) criteria for schizophrenia, mania, schizoaffective disorders, and bipolar disorders. Each consultation lasts 10 to 25 min, which results in approximately 12 h of talk. At the time of data-gathering, psychiatrists and their patients were engaged in an ongoing relationship that had lasted from six months to five years. In the routine consultation, regular meetings took place approximately once a month.

The audio recordings and the transcripts were analyzed using conversation analysis which takes the authentic, naturally occurring talk-in-interaction as data and seeks to explore the sequentiality of social actions (Schegloff, 2007). That is, it not only explores the current turns of talk, but also the related turns preceding and after the current turn. The data were transcribed according to the transcription system developed by Jefferson (2004) (Transcription notations see Appendix). All identifying information has been changed to secure the privacy of all the participants.

The transcript has three lines; the first line is Chinese *Pinyin*, which presents the original data that the analysis is based on; in the second line, the utterance is glossed word by word to help the English-speaking readers know what is happening in the Chinese original; in the third line, the idiomatic translation of the Chinese original is provided. Prosodic and phonetic features like pauses, length of silence, stress, intonation, and vocalic lengthening were also transcribed. Besides, the punctuation symbols in the transcription are not used grammatically, but to indicate the intonation contours of talk-in-interaction.

In order to explore how the psychiatrists dealt with patients’ perspectives toward treatment through formulating, three steps were carried out in the present article. In the first step, all the formulation sequences were identified from the data at hand. The formulation in the study is understood according to Heritage and Watson’s (1979) definition: an utterance that summarizes or paraphrases the understanding of the previous speaker’s talk while introducing an altered version of it. The formulation makes the patient’s confirmation or disconfirmation relevant. In addition, considering continuity of care across encounters is the nature of routine psychiatric consultation, we have included formulations of patients’ treatment preferences expressed in previous encounters and patients’ treatment behaviors, including their adherence/nonadherence to the prescribed treatment. The search identified 39 such instances. In the second step, a sub-collection of the sequences in which the patients expressed their treatment perspectives and the psychiatrist formulated the perspectives was formed. There were 22 instances of such formulations. The rest of them (17 instances), which were not studied in the study at hand, were mainly found in the interactional context where the psychiatrists reviewed the patients’ daily life experiences. In the third step, the 22 instances were analyzed in the local context of interaction. Specifically, three aspects were examined, including the patients’ treatment preferences, the design features of psychiatrists’ formulation, and the turns and agendas that follow the formulation.

## 4. Results

Through observation and analysis of the data, we found that the psychiatrists used two different types of formulations to attend to the patients’ treatment preferences. Specifically, when there were opposing stances (Stivers, 2008; Landmark et al., 2016), the psychiatrists formulated the patient’s stance as less than fully legitimate, challenging legitimacy of the patients’ perspective and indirectly conveying the stance preferred by the psychiatrists themselves (this occurred in 14 out of 22 cases); when there was a lack of opposing stances, the psychiatrists, through formulation of the patient’s congruent stance, checked and established legitimacy of patients’ attitudes toward treatment, preparing the ground for a particular treatment decision (in 8 cases). Table 1 presents the types and frequency of these formulations and describes their sequential environment.

**Table 1:** Description of the two types of formulation.

Types of formulation	Number	Frequency	Sequential environment
Formulation for establishing legitimacy of patient’s stance	8	36.4%	Where patients and psychiatrists had congruency on treatment preferences
Formulation for challenging legitimacy of patient’s stance	14	63.6%	Where patients and psychiatrists had opposing stances on treatment preferences
Total	22	100%	

We will begin with formulation sequences that establish legitimacy of patients’ perspectives toward treatment. The four data extracts that are discussed in the following are the most illustrative and representative of the variation within the categories.

### 4.1. Establishing legitimacy of patients’ treatment preferences

In sequences where patients and psychiatrists had congruency on treatment preferences, the psychiatrists highlighted the congruent aspect and established legitimacy of the patients’ stance. Extract 1 contains an example. Right before the extract, the patient, a 23-year-old woman diagnosed with mania, is reporting to the psychiatrist her recent health condition, which goes *very stable* (data not shown). In all data extracts, psychiatrists are referred to as Psy and patients as Pat. The turns that contain the psychiatrists’ formulations are indicated by arrows.

#### Extract 1

1. Pat: daifu ni kan e zhe ci neng jian jian yao ma?
  doctor you see er this time can decrease decrease drug QM
  Doctor, could the dosage be decreased this time?
2. Psy: [wo kan kan]
  I see see
  [Let me see]
3. Pat: [wo ganjue wo] dou hao le ↑zhe:me chang↓ shijian le.
  I feel I already good PRT so long time PRT
  [I- I feel all] better for ↑su:ch a long time↓
4. →Psy: hehe zuijin huifu hai bu [cuo,
  ((laughter)) recently recover still N bad
  Heheh your condition is not bad [recently,
5. Pat: [en
  [Yeah
6. → Psy: zai bu. jian- zai bu. jian,
  if N reduce if N reduce
  If-if the dosage could not be reduced this time,
7. → jiu (0.2) shiqu xinxin le ha=
  just lose heart PRT ((laughter))
  You would (0.2) be discouraged and disappointed=
8. Pat: =en hehe
  =Yeah heheh
9. (0.3)
10. Psy: hao, gei dian xiwang.
  OK give some hope
  OK, I will make you encouraged.
11. Pat: ⸰hehe⸰
  ⸰Heheh⸰
12. Psy: xian e ba zhe ge- zhe ge kuiliuping jian jian liang.
  first er ASV this CL this CL Quetiapine reduce reduce dose
  I am going to er start from reducing the-the dose of Quetiapine.
  ((Psy continues to introduce the regimen of Quetiapine decrease.))

After description of her *very stable* condition, the patient in line 1 makes a request, proposing a dosage decrease and explicitly soliciting the psychiatrist’s grant to the requirement. In line 3, the patient restates her current condition, which can be understood as an account for her request. In her account, the overlapped turn (indicated by the square brackets), the lengthened *su:ch*, the emphasized *long* (indicated by the underline on the transcript) together with a rising-falling intonation in line 3 seem to display the impatience and disappointment from the patient, implying her strong wish to reduce the dosage.

The psychiatrist seems to get the patient’s emotional state (see Weiste, 2016) as well as her strong wish. In line 4, he formulates the patient’s health condition as *not bad* and then formulates the patient’s treatment preferences in lines 6–7, providing something implied but unsaid (Bolden, 2010) in the patient’s previous talk, that is, the patient would be *discouraged and disappointed if the dosage could not be reduced this time*. This formulation is immediately confirmed by the patient in line 8. The laughter in line 7 is worth noticing. Previous studies have revealed that in medical settings, laughter is most often considered as a socially valuable phenomenon (Zayts and Schnurr, 2011) and is usually connected with a bonding social experience (Haakana, 2002). In line with the previous research, when the psychiatrist formulates the patient’s emotional state in lines 6–7, he infuses his expression with laughter, which appears to be a way to enacting the therapeutic principle of congruence and contributing to an effective psychiatrist-patient relationship.

In this sequence, the psychiatrist checks his understanding of patient’s stance toward treatment and establishes legitimacy of the stance through formulation. In the turns that follow, the psychiatrist grants the request before the discussion concerning the regimen of the Quetiapine decrease.

Extract 2 is another example that contains formulating patients’ stance as congruent. In the extract, the patient, a 38-year-old man diagnosed with schizophrenia, is reporting to the psychiatrist his recent health condition. Different from the patient in Extract 1, the patient in this extract does not have a completely congruent stance with the psychiatrist. In spite of this, the psychiatrist finds some congruent aspect and eventually constructs a congruent stance through formulation.

#### Extract 2

1. Pat: shengyin jibenshang ting bu dao le,
  voice basically hear N RVC PRT
  The voice is basically gone,
2. jiushi: e chi le zhe ge yao ba,
  but er have PRT this CL drug PRT
  Bu:t er after I took the drug,
3. Psy: ⸰en⸰
  ⸰Hm⸰
4. Pat: .hh jiu juede hun shen mei jin.
  just feel all over body N strength
  .hh I feel sluggish.
5. → Psy: na ni juede wenti zai zhe ge xin jia de zhe ge yao shang¿
  so you think problem PR this CL newly add NOM this CL drug PR
  So you think it is likely to be due to the-the drug prescribed last time¿
6. Pat: dui, chi le zhihou fanzheng jiu shi e
  yes take PRT after anyway just be er
  Yes, after taking it anyway I just er
7. baitian mei jing[shen wanshang hai shui bu. zhao,
  daytime N cheer up night moreover sleep N RVC
  feel dozy in the [daytime and hard to get sleep at night,
8. → Psy: [danshi shengyin-huanting queshi hao duo le.
  but voice hallucination indeed good much PRT
  [But the voice-hallucination is indeed lessened much
9. Pat: dui suoyi jiu- daifu nin kan kan gai zenme nong yixia,
  right so just doctor you see see should how do CSC
  Yes, so I will-doctor what should we do,
10. hai pa- hai pa ba gei qu nian side=
  and afraid and afraid PRT PR last year like
  and I am afraid-afraid of what happened last year=
11. Psy: =suoyi shuo zhe ge yao,
  so say this CL drug
  =So this drug,
12. zan hai bu. gan nage suibian tiao suibian jian,
  we still N dare that randomly adjust randomly reduce
  It is risky to er lower the dosage back down,
13. Pat: ⸰en⸰
  ⸰Yeah⸰
14. Psy: zai fan lou.
  in case relapse PRT
  for fear of the coming back ((of the schizophrenic symptoms))
15. Pat: en jiu shi a.
  hm just be PRT
  Yes, it is.
16. Psy: zhe yang,
  this way
  Let us do like this,
17. ba yong yao shijian tiao yixia,
  ASV take drug time adjust CSC
  rearranging the times of daily drug taking,
18. (0.3)
19. liang bu bian.
  dose N change
  without changing the total dose.
  ((Psy continues to introduce the details of the rearrangement.))

During the 2 min before the above extract starts, the patient provided a description of his recent health condition as a response to the psychiatrist’s inquiry. In lines 1–4, he continues reporting that the voice that used to haunt him has basically disappeared, but he feels sluggish after taking the drug, in this case, Amisulpride (data not shown), which appears to indicate that his being sluggish is due to the drug. Reporting a medication side effect problem may be understood as a request for medicine change or even for elimination of the drug altogether (Angell and Bolden, 2015; Bergen et al., 2018). In line 5, the psychiatrist produces an upshot formulation initiated with *so*, offering his version of the patient’s previous talk. The turn-initial particle so (*Na* in Chinese) shows that the formulation is a valid understanding inferred from the previous talk, but it transforms the frame of the talk by highlighting the potential reason for the patient’s sluggish condition, explicitly attributing it to the drug (i. e. the Amisulpride) prescribed during last visit.

After an immediate confirmation, the patient further explains in detail his sluggish condition: *dozy in the daytime but hard to get sleep at night*. Further, he emphasizes that these symptoms appear after he takes the medicine (indicated by the underline on the transcript). Up to now, it seems that they have a similar attitude toward the drug, i.e., it caused the side effect.

However, the psychiatrist’s formulation in line 8 transforms the direction. By highlighting therapeutic effects of the drug, the psychiatrist transforms the stance toward it, that is, from a negative attitude to an obviously positive one. Thus, the formulation in line 8, together with the one in line 5, characterizes the drug as having a Janus-faced feature. Further, by adding the adverbs *indeed* and *much*, the psychiatrist puts the therapeutic effect of the drug before its side effect, thus constructing a stance toward this drug, i.e., it causes some side effects but does lessen the patient’s symptoms.

In line 9, the patient confirms the stance and he seems to be going to provide some solutions but then cuts himself off before he turns to the psychiatrist for help. He further consolidates the trickiness of the situation by mentioning his seemingly troubling history (the coming back of the schizophrenic symptoms), which is echoed by the psychiatrist in the following turns. The patient in line 15 shows his affiliation with the delicate situation.

In this sequence, the psychiatrist step by step establishes a congruent stance with the patient, preparing the grounds for a decision, that is, neither to change the drug nor to reduce the dose of it but to modify the times of daily drug taking. So when this suggestion is brought up, it is presented and accepted as a straightforward course of action.

In sum, we found that when there were congruent stances, the psychiatrists highlighted the congruency; when there were not completely congruent stances, the psychiatrists would nonetheless find the congruent aspect and attempt to construct a congruent stance. After establishing legitimacy of the stance, the psychiatrists would engage the patients in negotiating enactment of a particular treatment decision.

### 4.2. Framing patients’ treatment preferences as illegitimate

In the data, we found that when there were opposing stances, the psychiatrists may frame the patient’s stance as less than fully legitimate work (Landmark et al., 2016) and thus constructed the patient’s stance as in opposition to treatment decisions proposed by the psychiatrists. In Extract 3, the psychiatrist opens the encounter by reviewing the patient’s medical records and then he points out that the patient did not follow the instructions on medication taking.

#### Extract 3

1. Psy: ni zhe- wo shi shang yue e sihao gei ni kai de yao,
  you this I be last month er fourth PR you prescribe NOM drug
  You are-I prescribed the drugs e:r the 4th last month,
2. zenme hai sheng zheme duo¿
  why still left so many
  Why are there so many left¿
3. Pat: e en wo- wo nage chi le dian zhongyao.
  er hm I I that have PRT some traditional Chinese medicine
  Er hm I- I took some traditional Chinese medicine.
4. → Psy: ni ba wo gei ni kai de yao ting le,
  you ASV I PR you prescribe NOM drug stop PRT
  So you stopped taking the drugs I prescribed,
5. → you cong biede difang na de zhongyao (0.4)
  then from other place buy NOM traditional Chinese medicine
  then took some traditional Chinese medicine (0.4)
6. Psy: shi ba?
  be QM
  Right?
7. Pat: °en°
  °Hm°
8. Psy: ni zhe bing hai zaozhe ne,
  you this illness still far PRT
  You are far from being fully recovered,
9. ni zenme neng ting yao a¿
  you how can stop drug PRT
  How can you stop taking the drugs¿
10. Pat: wo yiwei-wo yiwei chi chi zai dui shenti bu. hao,
  I think I think have have may PR body N good
  I think- I think the drugs are harmful to my body,
11. na yao xishou bu. liao a an juede.
  that drug absorb N RVC PRT I think
  They cannot be absorbed, I think.
12. renjia zhongyao daifu shuo
  that traditional Chinese medicine doctor say
  That traditional Chinese medicine doctor said
13. xishou bu liao.
  absorb N RVC
  they cannot be absorbed.
14. Psy: aiya, zhongyao daifu na-
  gee traditional Chinese medicine doctor that.
  Gee, the traditional Chinese medicine doctor-
15. ta na ge xishou hao,
  he that CL absorb good.
  even if his medicine can be absorbed well,
16. gei ni zhi le bing le mei you a?
  for you cure RVC illness PRT N have PRT.
  has it cured your symptoms?
17. (0.3)
18. Psy: en ha?
  Hm ha?
19. Pat: wo juede zhe ge yao fu zuoyong tai da le.
  I think this CL drug side effect too big PRT
  I think these drugs have many side effects.
20. Psy: na ge yao dou shi- shi yao sanfen du.
  any CL drug all be be drug some poison
  Any drug is- every drug has side effects.
21. Pat: en
  Hm
22. → Psy: suoyi shuo ni bu. neng-
  so say you N can
  So you cannot-
23. → ni zhe jiao yinyefeishi a.
  you this call giving up eating for fear of choking PRT
  what you are doing is giving up eating for fear of choking.

The patient in this extract, a 34-year-old woman, has been diagnosed as having schizophrenia and this is one of her routine follow-up visits. From the transcript, we learned that according to the prescribed treatment plan, she should have finished the drugs prescribed by the psychiatrist right before this visit. However, the psychiatrist finds out that the patient did not follow the instructions and has stopped taking the drugs.

The target lines in this extract are two instances of formulation in lines 4–5 and line 23. On the basis of the question-answer sequence in lines 1–3, the psychiatrist, in lines 4–5, makes a formulation of the patient’s nonadherence behavior toward the prescribed treatment, that is, she stopped taking the prescribed drugs and took some traditional Chinese medicine instead. Such kind of formulation is a B-event statement (Labov and Fanshel, 1977) about which the patient has primary epistemic authority (Heritage, 2013) and is treated as requests for confirmation. Indeed, the patient provides confirmation in line 7, although it is only a minimal one, *hm* in this case, in a soft voice.

In line 4, the psychiatrist points out that the patient has stopped taking the prescribed drugs. The particle *ba* (active syntactic verb marker in Chinese) highlights the patient’s stance as active, that is, she has actively turned down the previously agreed treatment recommendations. The subsequent formulation in line 5 articulates what the patient has done: *took traditional Chinese medicine instead*.

The formulation in lines 4–5 puts the patient’s stance on the table (Heritage, 1985) and it is taken as a basis for negotiating the illegitimacy of the patient’s stance. In line 8, the psychiatrist states that the patient is far from being fully recovered, which may imply the inappropriateness of the patient’s nonadherence behavior. In line 9, he further produces an unanswerable question *how can you stop taking the drugs* that conveys a challenging stance and further highlights the wrongness of the patient’s nonadherence. However, it is noticeable that the patient’s response (lines 10–13) to the question is interesting. In line 10, the patient responds by saying that she thinks the drugs may have some side effects and may be harmful to her body. Then she reinforces her view by stating that the drugs cannot be absorbed in line 11. In lines 12–13, the patient further justifies her views by distributing the responsibility to another medical authority, *the doctor of traditional Chinese medicine*.

In response to the patient’s justification, the psychiatrist challenges her notion by producing a rhetorical question: *even if the traditional Chinese medicine can be absorbed well, has it cured your symptoms*? (lines 14–16). He then continues to state that every drug has some side effects, which leads to his formulation in line 23: *what you are doing is giving up eating for fear of choking.* Prefaced by *so* (*suoyishuo* in Chinese), a canonical formulation marker, this formulation can be taken as a concluding evaluation of the patient’s stance and the use of the Chinese idiom *giving up eating for fear of choking* (yinyefeishi in Chinese) further frames the patient’s stance as illegitimate.

It is noted that the psychiatrist’s formulations of the patient’s nonadherence behaviors and views (lines 4–5 and line 23) not only bring up the patient’s treatment preferences and make her perspective relevant, but may also function as a device for evaluating and challenging legitimacy of the patient’s treatment preferences, preparing grounds for negotiating other treatment options. First, they manage to challenge the patient’s implausible views and treatment behaviors. The psychiatrist uses them as a starting point for framing her stance as not in accordance with the medical professionals and negotiating for opposing treatment options. Secondly, they are employed to put the patient’s stance on the table and make it an active wish, thus framing the patient as an active participator who is projected as accountable for the treatment (Thompson and McCabe, 2018).

It is interesting that in the process of treatment decision making, when the psychiatrist framed the patient as a responsible agent, sometimes the patient would show resistance to the agency. For instance, in lines 12–13, in the patient’s justification of her treatment views and behaviors, it seems that she is reluctant to take more agency by introducing an authoritative third party, i. e. *the doctor of traditional Chinese medicine*.

Extract 4 provides another example. In this extract, the patient, a 36-year-old man who is suffering from schizoaffective disorder, is discussing with his psychiatrist about the treatment recommendation. Before the extract (data not shown), through the conversation with the patient and also referring to the report from the man’s parents, the psychiatrist learned that the patient’s condition was worsened recently and he suggested that the patient should be hospitalized. His parents supported the proposal but the patient did not. The extract starts when the psychiatrist formulates the patient’s stance toward hospitalization.

#### Extract 4

1. → Psy: ni fumu hai shi xiang rang ni zhuyuan,
  you partent still be will let you hospitalize
  your parents also want you to be hospitalized,
2. → danshi ni bu. yuanyi zhu¿
  but you N will hospitalize
  But you do not want to¿
3. Pat: en
  Hm
4. Psy: neng gaosu wo weishenme ma?
  can tell me why QM
  Could you tell me why?
5. weishenme bu yuanyi zhu¿
  why N willing hospitalize
  Why do not you want to be hospitalized¿
6. Pat: e: guanjian shi wo- wo ye bu. shi shuo wo jiu shi feng le shenmede,
  er point be I I also N be say I just be mad PRT whatever
  E:r the point is I-I am not mad or something like that,
7. wo yiqian kan de daifu,
  I before see NOM doctor
  I’ve seen many doctors,
8. dou mei shuo guo rang zhuyuan.
  all N say ASP let hospitalize
  no one of them asked me to be hospitalized.
9. → Psy: ni hai bu. juede ni zhe shi bing?
  you still N think you this be illness
  you have not been aware of your illness?
10. → dou shi lai nian le.
  all ten about year PRT
  even you have suffered from it for about 10 years.
11. Pat: wo jiu shi juede ba wo mei name yanzhong.
  I just be think PRT I N that serious
  I just do not think my problem is that serious.

The patient in the extract is a 36-year-old man who is suffering from schizoaffective disorder for about 10 years and the situation has been gradually worsened recently. After evaluation of the patient’s condition (data not shown), the psychiatrist proposed him to be hospitalized but he said *no* to the proposal even though his patients agreed on the hospitalization treatment. In lines 1–2, the psychiatrist formulates the patient’s stance toward the hospitalization treatment. By introducing the opinion of a significant third party (his parents in this case), the psychiatrist seems to indicate that the patient’s stance is not implausible. After a confirmation from the patient in line 3, the psychiatrist enquires about the reason through a rhetorical question *Why do not you want to be hospitalized* in line 5. Such questions treat the patient as not in accordance with common sense, thus conveying a challenging stance.

In line 6, the patient justifies his refusal by stating that *I am not mad or something like that*, then he further makes a justification by introducing some authoritative third party, i.e., many other doctors. In response to the patient’s justification, the psychiatrist in lines 9–10 produces another formulation: *you have not been aware of your illness even you have suffered from it for about 10 years*, which challenges the patients’ poor awareness of his own problems. Note that the rhetorical question *you have not been aware of your problem*, together with the turn increment *even you have suffered from it for about 10 years*, especially the adverb *even*, displays the absurdity of the patient’s views and challenges his stance. The patient’s elaboration in the next turn suggests the poor awareness of his illness, which is one of the factors resulting in treatment resistance among patients with severe mental disorders (Quirk et al., 2012).

To summarize, when there are opposing stances, the psychiatrists would, on the one hand, formulate patient’s implausible views or behaviors toward treatment and delegitimize the patient’s stance; on the other hand, by formulating patient’s perspective as active treatment preferences, they would frame the patient as an active agent who is projected as accountable for his/her stance. In this way, the psychiatrists challenged the patient’s stance and indirectly conveyed their own opposing stance toward the treatment.

## 5. Discussion and conclusion

In this article, we have scrutinized how – and in what interactional contexts – the psychiatrists dealt with patients’ stance and perspectives toward treatment by issuing formulations of what the patients have said in relation to treatment. Specifically, the psychiatrists attended to patients’ treatment preferences differently depending on whether there were opposing stances (Landmark et al., 2016) toward treatment. When the psychiatrists and patients had congruent stance, the psychiatrists would highlight the congruency (as in Extract 1); even if there was not a completely congruent stance, the psychiatrist would nevertheless find or construct some congruent aspect through formulating patients’ stance (as in Extract 2). In both cases, formulation may be used by the psychiatrists to elicit and check patients’ views and preferences, fostering mutual understanding and preparing grounds for a specific treatment recommendation. When there were opposing stances, the psychiatrists formulated the patients’ stance as less than fully legitimate, thus challenging legitimacy of the patients’ perspectives and indirectly conveying the stance preferred by the psychiatrists (as in Extract 3 and 4). This is in accordance with the challenging formulation found in cognitive behavioral therapy, through which the therapist transforms the client’s talk into something that is implausible, thus challenging the client’s non-agentic position (Yao et al., 2022).

Further, we found that in the cases of congruence (as in Extract 1 and 2), the patient operated from the beginning within a medical frame, i.e., weighing the pros and cons of medication regarding treating the symptoms and the side effects. When the patient adhered to the medical framework, the psychiatrist picked it up, reformulated it and worked with the patient’s statements. In the first two extracts there is a sense of collaborative conversational practices because they both adhere to the medical frame. However, when the patient introduced a different frame (as in Extract 3 and 4), i.e., traditional Chinese medicine, the psychiatrist dismissed and challenged that frame and their statements came from within the medical frame, assuming expertise and authority. In the last two extracts there is much more a feeling of non-communication, because of lack of common ground, and the psychiatrist proffers statements from a position of authority.

Doctor authority is thought to be a Chinese cultural phenomenon that conventionally resulted in patient compliance in medical practice (Liu et al., 2015). The paternalistic approach has long been a dominant approach in Chinese medical culture. Under this approach, doctors are granted absolute authority to make treatment decisions, obviating the need for persuasion and justification from the doctors. Recently, under the influence of patient-centered ideology, the shared-decision making approach has gradually drawn attention from researchers and doctors (Yang et al., 2022). However, the previous studies show that although Chinese doctors and patients mostly acknowledge the advantages of shared-decision making approach, they admit that the diffusion of this model is rather limited in the medical practice (Zhang et al., 2020).

Our study shows that even though the psychiatrists are granted institutional authority to make treatment decisions, they routinely make use of formulations of patients’ stance to achieve consensus about the treatment plan. For instance, by indicating the illegitimacy of patients’ stance, the psychiatrists indirectly convey their opposing stance, ultimately aiming to pursue patients’ agreement on treatment decisions that are in accordance with their own professional opinions, while avoiding a more authoritarian approach. To some degree, the use of formulation indicates that everyday psychiatric practice might inhabit a middle ground between the paternalistic model and the patient-centered philosophy idealized in the model of shared decision making (Angell and Bolden, 2015). Our findings add to a growing body of literature suggesting that the shared decision-making model is only partially actualized in actual psychiatric practices (Quirk et al., 2012). The findings further suggest that there is still a long way to go from the standard psychiatric practices to the ideal model of shared decision making described in guidelines.

In addition, our findings display the interesting tension between traditional Chinese medicine and pharmacological approach in psychiatry (as in Extract 3). As part of traditional Chinese culture, traditional Chinese medicine has a history of more than 5,000 years. However, with the development of modern medicine in China, pharmacological approach is becoming dominant in psychiatry while traditional Chinese medicine is gradually marginalized mainly due to its lack of feasible criteria for diagnosis and treatment of mental disorders (Zhang, 2020). In this sense, this study provides a window through which we could have a better understanding of the complex nature of psychiatric treatment decision making in Chinese medical culture.

Note that although we have explored the formulation as a conversational practice to deal with patients’ stance, it is very likely that other conversational practices than the formulation may be used in treatment decision making in psychiatry. To have a deeper understanding of the process of decision making, increasingly more adequate documentation of them is necessary. Moreover, the use of audio recordings for exploring face-to-face interaction is limiting because the participants’ non-verbal conducts and embodied activities are unavailable to the analysts.

To conclude, this study documented how the psychiatrists seek and discuss patients’ treatment preferences in routine outpatient psychiatric care by issuing formulations of the patients’ talk in relation to treatment through fine-grained conversation analysis of naturally occurring, face-to-face psychiatrist-patient talk-in-interaction as it folds. The strength of the conversation analytic methodology adopted in this article lies in its efforts to explore in detail the conversational practices that bring the core elements of shared decision making into the process of decision making in authentic consultations. Previous conversation analytic studies on formulation in institutional settings have highlighted its functions to demonstrate in detail the interactional realization of the core objectives in a particular setting, such as how to do active listening in child counseling (Hutchby, 2005) and how to ascribe agency to the clients in cognitive behavioral therapy (Yao et al., 2022). This study contributes to this line of research by providing insight into how formulation of patients’ stance might achieve other objectives than merely eliciting and checking understanding of patients’ views and preferences toward treatment in routine outpatient psychiatric consultations. In this sense, the findings of the research may complement psychiatrists’ professional stock of interactional knowledge (Peräkylä and Vehviläinen, 2003) with which psychiatric consultations get accomplished.

Overall, the findings of this study have potential implications for medical education in general and for psychiatric practice and training in particular. For instance, they may make relevant applied work aimed at raising mental health professionals’ awareness of conversational practices they employ to deal with issues related to patient involvement, patient awareness and general debate on the mental health of people, particularly after COVID-19 lockdown.

## Data availability statement

The original contributions presented in the study are included in the article/supplementary material, further inquiries can be directed to the corresponding author.

## Ethics statement

Ethical review and approval was not required for the study on human participants in accordance with the local legislation and institutional requirements. The patients/participants provided their written informed consent to participate in this study.

## Author contributions

XY and XZ: conception and design of the study, acquisition, analysis, interpretation of data, and drafting and revising the manuscript. All authors contributed to the article and approved the submitted version.

## Funding

This research was funded by MOE (Ministry of Education in China) Project of Humanities and Social Sciences (No. 21YJC740074).

## Conflict of interest

The authors declare that the research was conducted in the absence of any commercial or financial relationships that could be construed as a potential conflict of interest.

## Publisher’s note

All claims expressed in this article are solely those of the authors and do not necessarily represent those of their affiliated organizations, or those of the publisher, the editors and the reviewers. Any product that may be evaluated in this article, or claim that may be made by its manufacturer, is not guaranteed or endorsed by the publisher.

## Appendix: Transcription notations

(0.2) A number inside brackets denotes a timed pause.

[] Square brackets denote a point where overlapping speech occurs (beginning [and end]).

_ When a word or part of a word is underlined, it denotes a raise in volume or emphasis.

:: Colons represent elongated speech, a stretched sound.

° ° When there are two degree signs, the talk between them is markedly softer than the talk around it.

. A full stop marks a falling intonation.

? A question mark marks a rising intonation.

, A comma marks a slightly rising intonation, but is also used to indicate ‘continuing’ intonation.

¿ An upside-down question mark is used for intonation which rises more than a slight rise (,) but is not as sharp a rise as for a question mark.

- A hyphen after a word or part of a word indicates a cut-off or self-interruption.

= Equals signs represent latched speech, a continuation of talk. They ordinarily come in pairs – one at the end of a line and another at the start of the next line or one shortly thereafter.

↑ ↓ The up and down arrows denote marked upstep/downstep in intonation.

. hhh Hearable in-breathing is shown by a dot before the “h,” − the more “hs,” the more in-breathing.

((description)) Double parentheses are used to mark transcriber’s descriptions of events, rather

than representations of them. Thus ((cough)), ((sniff)), ((telephone rings)), ((footsteps)), ((whispered)), ((laugh)), etc.

## Abbreviations

ASP: aspectual marker
ASV: active syntactic verb marker
be: BE verbs (shi)
CL: classifier
CSC: complex stative construction
N: negation
NOM: nominalizer (de)
PR: preposition
PRT: particle
QM: question marker
RVC: resultative verb complement
